# Laboratory evaluation of RealStar Yellow Fever Virus RT-PCR kit 1.0 for potential use in the global yellow fever laboratory network

**DOI:** 10.1371/journal.pntd.0010770

**Published:** 2022-09-06

**Authors:** Alison J. Basile, Matthias Niedrig, Amy J. Lambert, Robyn Meurant, Aaron C. Brault, Cristina Domingo, Christin H. Goodman, Barbara W. Johnson, Eric C. Mossel, Mick N. Mulders, Jason O. Velez, Holly R. Hughes

**Affiliations:** 1 Arboviral Diseases Branch, Division of Vector-Borne Infectious Diseases, Centers for Disease Control and Prevention, Fort Collins, Colorado, United States of America; 2 WHO consultant, Berlin, Germany; 3 ACT-IVD, Bougival, France; 4 Public Health Laboratory Support Unit, Centre for International Health Protection, Robert Koch Institute, Berlin, Germany; 5 Scientific Laboratory Consulting, Laporte, Colorado, United States of America; 6 World Health Organization, Geneva, Switzerland; Aix-Marseille Universite, FRANCE

## Abstract

**Background:**

Early detection of human yellow fever (YF) infection in YF-endemic regions is critical to timely outbreak mitigation. African National Laboratories chiefly rely on serological assays that require confirmation at Regional Reference Laboratories, thus delaying results, which themselves are not always definitive often due to antibody cross-reactivity. A positive molecular test result is confirmatory for YF; therefore, a standardized YF molecular assay would facilitate immediate confirmation at National Laboratories. The WHO-coordinated global Eliminate Yellow Fever Epidemics Laboratory Technical Working Group sought to independently evaluate the quality and performance of commercial YF molecular assays relevant to use in countries with endemic YF, in the absence of stringent premarket assessments. This report details a limited laboratory WHO-coordinated evaluation of the altona Diagnostics RealStar Yellow Fever Virus RT-PCR kit 1.0.

**Methodology and principal findings:**

Specific objectives were to assess the assay’s ability to detect YF virus strains in human serum from YF-endemic regions, determine the potential for interference and cross-reactions, verify the performance claims as stated by the manufacturer, and assess usability. RNA extracted from normal human serum spiked with YF virus showed the assay to be precise with minimal lot-to-lot variation. The 95% limit of detection calculated was approximately 1,245 RNA copies/ml [95% confidence interval 497 to 1,640 copies/ml]. Positive results were obtained with spatially and temporally diverse YF strains. The assay was specific for YF virus, was not subject to endogenous or exogenous interferents, and was clinically sensitive and specific. A review of operational characteristics revealed that a positivity cutoff was not defined in the instructions for use, but otherwise the assay was user-friendly.

**Conclusions and significance:**

The RealStar Yellow Fever Virus RT-PCR kit 1.0 has performance characteristics consistent with the manufacturer’s claims and is suitable for use in YF-endemic regions. Its use is expected to decrease YF outbreak detection times and be instrumental in saving lives.

## Author summary

Molecular diagnosis of human specimens is the fastest way to confirm and subsequently mitigate yellow fever (YF) outbreaks. In Africa, a serological method requiring lengthy confirmation external to the National Laboratories is standard and results are not always definitive due to factors such as cross-reactive antibodies. Molecular testing has been uncommon in African YF laboratories, largely due to challenges with acquiring reagents and a lack of an approved assay that simplifies laboratory procedures. In our capacities as members of the global Eliminate Yellow Fever Epidemics strategy, we independently evaluated the RealStar Yellow Fever RT-PCR kit 1.0 (altona Diagnostics, Hamburg, Germany). We showed that the assay is sensitive and specific for detection of RNA from representative YF virus strains found worldwide. It is not subject to interference from common blood contaminants and has good clinical performance. In conjunction with a separately conducted review of the manufacturer’s quality systems, we concluded that the RealStar Yellow Fever Virus RT-PCR kit 1.0 is appropriate for use in YF endemic regions. Logistical and funding support to introduce the assay is being arranged, which should decrease YF outbreak detection times and ultimately save lives.

## Introduction

Yellow fever (YF) virus, the prototype virus of the genus *Flavivirus*, is endemic in a total of 47 countries in tropical and subtropical regions of Africa and Latin America [1]. YF virus exists as a single serotype that encompasses seven major lineages with five from Africa [2] and two from South America [3]. Transmitted chiefly by *Aedes* and *Haemogogus* mosquito species, YF virus infects human and non-human primate populations [2]. The virus has a positive-sense, 11 kb single-stranded RNA genome that encodes three structural and seven nonstructural proteins, where the envelope protein is the primary immunogen [4]. The highly effective attenuated 17D vaccine has been used since 1937 [5].

Clinical YF disease may be self-limiting or progress to a severe form, with a case fatality rate of 30–60% for the latter [6]. The true burden of current YF disease is difficult to estimate, but major outbreaks in Angola, Democratic Republic of Congo, and Brazil in 2015–2019 have highlighted the inadequacies of vaccination coverage and over-extended vaccine supplies [7–9]. The judicious vaccination of populations at risk of, or experiencing, YF outbreaks is contingent upon many factors including history of vaccination campaigns, accurate clinical and laboratory diagnosis, and vaccine availability and funding. In 2017, the global Eliminate Yellow Fever Epidemics (EYE) strategy was initiated with the goal of eliminating YF epidemics and major outbreaks by 2026. The EYE strategy comprises a consortium of over 50 international organizations including the World Health Organization (WHO), United Nations Children’s Fund (UNICEF) and Gavi, the Vaccine Alliance [10].

Initiation of reactive vaccination campaigns is heavily reliant on laboratory data, due to ambiguous symptoms of early clinical YF presentation which can easily be confused with other diseases such as malaria or other arboviral diseases [11,12]. Early, reliable detection of YF outbreaks in National Laboratories (NLs) is critical to enable initiation of vaccination campaigns [13].

A comprehensive laboratory assessment to understand the true YF testing capacities, gaps, and needs of 25 African laboratories in countries at high risk of YF was undertaken in 2018 as part of the EYE initiative [13]. Part of this initiative included a survey of molecular testing capacities. At this time, a molecular component had only recently been incorporated into the African YF testing algorithm [14]. The region still relied almost exclusively on serological testing; however, the laboratory assessment revealed that 22 NLs had equipment and staff trained in molecular testing for viral nucleic acids, and that by 2018, 10 of these labs were performing YF RT-PCR [13]. Conversely, molecular assays in Latin America were already well-established and supported by the Pan American Health Organization (PAHO) using the method of Domingo et al. (2012) [15].

A positive molecular result is confirmatory for diagnosis of YF in the absence of YF vaccination in the previous 7 days and is most likely to be obtained from specimens taken within 10 days of symptom onset [14]. By contrast, front-line serologic immunoglobulin M testing methods [16,17] require confirmation and differential diagnosis by the resource-intensive plaque-reduction neutralization test (PRNT), typically only performed at Regional Reference laboratories (RRL) [18]. The necessary specimen transport between NL and RRL delays confirmatory testing. Even then, serological confirmation is often confounded by heterotypic antibodies found in secondary flavivirus infections [19]. The gaps identified by the referenced laboratory assessment were used to inform training and capacity-building. Workshops in Africa in 2019 included instruction on the YF molecular detection method widely-used in the Americas and clinically validated during the Brazilian YF outbreaks [20]. Follow-up practice panels distributed to the participating laboratories showed considerable inconsistency among results, indicating the need for further technical assistance. The use of kit-based commercial assays would simplify laboratory procedures and reduce reagent acquisition bottlenecks particularly in the African region.

In the absence of YF RNA detection assays approved for clinical use by a rigorous regulatory body, the EYE Laboratory Technical Working Group (LTWG) sought to independently evaluate commercially available YF molecular assays to investigate quality and performance. The independent evaluation comprised two parts: a) an assessment of the manufacturer’s quality system by an independent quality consultant and a review of manufacturer-provided performance data for the assay; and b) an independent laboratory performance evaluation of the product by a WHO Collaborating Center laboratory with documented regulatory standards. To this end, an announcement for Expressions of Interest (EOI) was published on the United Nations Global Marketplace website in 2020 [21]; wherein interested manufacturers of YF molecular assays were invited to subject their product for evaluation. If the outcome was successful, the product would be recommended to UNICEF for potential inclusion in the catalog and a request would be made to Gavi, the Vaccine Alliance, to support the procurement of the assay.

The United States Centers for Disease Control and Prevention (US CDC) Arbovirus Diseases Branch, a WHO Collaborating Center and Global Specialized Laboratory for YF, was chosen to perform the laboratory portion of the evaluation. This report details the laboratory evaluation of the RealStar Yellow Fever Virus RT-PCR kit 1.0 with the specific objectives of assessing detection of wild-type YF virus strains from Africa and South America, the potential for interference and cross-reactions within the assay, to verify the performance claims stated in the Instructions for Use (IFU) or in the manufacturer-provided performance data, and usability of the assay.

## Materials and methods

### Ethics statement

Human serum specimens used in this study were archived residual volumes from specimens originally collected for routine diagnostic activities at the US CDC (Atlanta, GA and Fort Collins, CO) and at Centre Pasteur du Cameroun (Yaoundé, République du Cameroun). All specimens were de-linked from any personal identifiers prior to the commencement of the study and prior to receipt at the CDC. The use of these specimens was approved under the CDC IRB protocol number 6773, USE OF HUMAN SPECIMENS FOR LABORATORY RESEARCH (2021).

### WHO evaluation protocol

The methods described here are outlined in the protocol WHO PROTOCOL FOR THE PERFORMANCE EVALUATION OF YELLOW FEVER Virus (YFV) MOLECULAR ASSAYS (S1 Protocol) and were generally adhered to, except as noted below.

### Assay under evaluation

RealStar Yellow Fever Virus RT-PCR kits 1.0 were provided by altona Diagnostics GmbH (Hamburg, Germany) at no cost to US CDC for this evaluation. Testing was performed in December 2020 using kits labeled with an expiration date of 28^th^ February 2021, and were stored at -20°C according to the manufacturer’s instructions prior to use.

### RNA extraction

RNA was extracted using the QIAamp Viral RNA Mini Kit (QIAGEN GmbH, Hilden, Germany) according to the manufacturer’s standard instructions using 140 μl specimen volume and a 60 μl elution volume for all parts of the evaluation except analytical sensitivity and precision measurements. For these, viral stocks of approximated RNA quantity were spiked into 100 μL of normal human serum. The entire 100 μL of spiked specimen was added to 400 μL of AVL buffer and incubated for 10 min at room temperature (22–25°C). After inactivation, 400 μL of 100% ethanol was added, and the mixture applied to spin columns and washed according to the manufacturer’s protocol. The RNA was eluted in 100 μL of AVE buffer to ensure the appropriate amount of quantified RNA was tested. Extractions were performed concurrently for all specimens, and extracts were frozen at -80°C until used with the RealStar Yellow Fever Virus RT-PCR kit 1.0.

### Real-time RT-PCR

RT-PCR data were generated for the specimens on the ABI Prism 7500 Fast SDS (Applied Biosystems, Inc, Waltham, MA), unless otherwise specified. External medium and low copy number quality controls, a negative extraction control from negative serum, and a no template control were included on each plate along with the positive control provided in the kit. Prior to performing the tests in the evaluation, all three operators passed a blinded proficiency test using the RealStar Yellow Fever Virus RT-PCR kit 1.0. The IFU was followed with no modifications, where 10 μl of extracted RNA was used per reaction.

### Intra-assay precision

Contrived specimens were created using normal human serum (NHS), (EMD-Millipore, Austin, TX) spiked with YF vaccine strain (17D-204) and two prototype strains, 614819 (Panama 1974), and Asibi (Ghana 1927). Relative standards from Quality Control for Molecular Diagnostics (QCMD, Glasgow, Scotland) were used to approximate the RNA concentrations at high, medium, and low RNA copy numbers [approximately 1,000,000 copies/ml, 100,000 copies/ml, and 10,000 copies/ml, respectively]. Extracted RNA was tested in quadruplicate by a single operator, and percent coefficients of variation around the mean (%CV) of cycle thresholds (Ct) were calculated as standard deviation divided by mean.

### Inter-assay precision

Three operators performed precision testing on three different days. Specimen RNAs were extracted from NHS that contained YF vaccine strain (17D-204) and two prototype strains, 614819 (Panama 1974), and Asibi (Ghana 1927), at high, medium, and low concentrations. These RNAs were tested in quadruplicate by each operator and %CVs were calculated.

### Inter-lot precision

Specimen RNAs were extracted from NHS that contained YF vaccine strain (17D-204) and two prototype strains, 614819 (Panama 1974), and Asibi (Ghana 1927), at high, medium, and low RNA concentrations. These RNAs were tested in quadruplicate by a single operator using each of two production lots of RealStar Yellow Fever Virus RT-PCR kits 1.0 and %CVs were calculated.

### Analytical sensitivity (Limit of Detection, LOD)

Relative standards from QCMD were used to approximate the RNA concentrations in a YF17D-204 dilution series. The reference assay of Domingo et al, (2012) [20] was used to verify the deduced copy numbers in the series. Five replicates each of a series of 2-fold dilutions were tested using the RealStar Yellow Fever Virus RT-PCR kit 1.0 as prescribed in the evaluation protocol. An additional series was created with five replicates each of lower dilutions to reach a better determination of LOD, three dilutions of which overlapped the original dilution series to ensure reproducibility. This effectively created a series of nine 2-fold dilutions. Final dilutions ranged from 25,000 copies/ml to 97 copies/ml where the three dilutions that overlapped (3125, 1562 and 781 copies/ml) had 10 total replicates and the others had five. The percent positive (detected) wells were plotted on a non-linear regression using a Sigmoidal, 4-parameter curve. Constraints were set to 100% and 0% to reflect boundaries of data. The concentration with 95% detection was interpolated.

### Inclusivity

RNAs extracted from nine YFV strains at high, medium, and low copy number concentrations were tested in quadruplicate by one operator using the RealStar Yellow Fever Virus RT-PCR kit 1.0 to evaluate inclusivity. Older and modern strains from the Americas and Africa were used to represent a diversity of origins and eras: 17D-204 (Vaccine strain), Asibi (Ghana, 1927), 614819 (Panama, 1974), BA-55 (Nigeria, 1986), BC-7914 (Kenya, 1993), 14FA (Angola, 1971), FMD-1240 (Peru 2007), CAREC M209 (Trinidad 2009), InHRR 10a-10 (Venezuela 2010).

### Analytical specificity: Cross-reactivity

A total of 15 non-YF viruses, including flaviviruses as well as other viruses that can occur in the same geographic region as YFV, were tested in singlet to evaluate analytical specificity using the RealStar Yellow Fever Virus RT-PCR kit 1.0. High RNA copy number Zika, West Nile, dengue serotypes 1–4, Powassan, Japanese encephalitis, chikungunya, Ebola, Marburg, Lassa, measles, HIV, and Influenza A H1N1 viruses were tested. To avoid RNA degradation, all virus samples were extracted directly from the source material and tested without the repeated extraction from serum, which was a deviation from the guiding document.

Hepatitis C, hepatitis E, and tick-borne encephalitis viruses were listed in the guiding document but with the consent of the EYE LTWG, were not tested in the evaluation.

### Analytical specificity: Interfering substances

A true malaria antigen-positive specimen, plus specimens contrived using NHS containing potentially interfering substances were used to determine whether these substances affected the detection of YF RNA. Serum containing hemoglobin (200 g/L), triglycerides (5.6 mM), bilirubin (257 uM), and malaria antigen were spiked with YF 17D-204 virus possessing approximately 25,000 copies/ml of RNA. RNAs extracted from these sera were then tested in triplicate by a single operator using the RealStar Yellow Fever Virus RT-PCR kit 1.0.

### Clinical performance using surrogate specimens

RNAs were extracted from 12 surrogate positive sera (10 negative clinical serum specimens from Cameroon spiked with YF strain Asibi (Ghana 1927) virus possessing approximately 25,000 copies/ml of RNA and 2 negative specimens from Ecuador spiked with YF virus strain 614819 (Panama 1974) possessing approximately 25,000 copies/ml of RNA. RNAs were also extracted from 45 negative sera (40 from Cameroon, 5 from Ecuador). The 12 virus-spiked specimen RNAs were tested using the RealStar Yellow Fever Virus RT-PCR kit 1.0 to approximate clinical sensitivity in place of true clinical specimens which were unavailable, and the negative sera from Africa and the Americas were used to assess clinical specificity in specimens from YF endemic regions. This evaluation deviated from the evaluation document in which an additional 43 true negative sera from South America were prescribed to make a total of 100 specimens. These could not be obtained due to competing priorities (i.e., COVID-19 pandemic).

### Platform comparison

A limited side-by-side precision evaluation of the Bio-Rad CFX96 instrument (Bio-Rad Laboratories, Hercules, CA) compared to the ABI Prism 7500 Fast SDS was performed. RNAs from YF vaccine strain (17D-204) and two prototype strains, 614819 (Panama 1974), and Asibi (Ghana 1927), at high, medium, and low concentrations were tested in quadruplicate by a single operator and %CVs were calculated.

### Operational characteristics of the RealStar Yellow Fever Virus RT-PCR kit 1.0

Operational characteristics of the assay were assessed during the evaluation to ensure they were compatible with desired features as listed in the target product profile (TPP) for YF molecular assays developed by the Foundation for Innovative New Diagnostics (FIND, Geneva, Switzerland) [22]. The IFU was evaluated for ambiguities, and for appropriateness for use in NLs in YF endemic regions.

## Results

### Intra-assay precision

Of the three virus strains tested, %CVs of Ct values were generally lowest for the YF17D-204 dilutions, ranging from 0.17 to 0.59%. Percent CVs were higher for other prototype strains (614819, range 0.41 to 2.49%; Asibi, range 0.57 to 3.19%). For all strains tested, the highest %CVs were observed in specimens possessing the lowest RNA concentrations (Table 1).

**Table 1:** Intra-assay precision of RealStar Yellow Fever Virus RT-PCR kit 1.0.

YF Strain	RNA copies/ml^a^	1	2	3	4	%CV
**17D-204**	**10** ^**6**^	28.3^b^	28.3	28.1	28.3	0.27
**(Vaccine)**	**10** ^**5**^	31.2	31.1	31.2	31.1	0.17
	**10** ^**4**^	35.1	35.4	35.0	34.9	0.59
**614819**	**10** ^**6**^	25.9	25.9	25.7	25.8	0.41
**(Panama 1974)**	**10** ^**5**^	29.0	29.9	29.8	29.8	1.45
	**10** ^**4**^	31.5	32.7	31.3	30.9	2.49
**Asibi**	**10** ^**6**^	28.4	28.4	28.2	28.1	0.57
**(Ghana 1927)**	**10** ^**5**^	31.4	31.3	30.2	31.3	1.80
	**10** ^**4**^	33.0	34.8	34.7	32.7	3.19

YF, yellow fever; RNA, ribonucleic acid; %CV, coefficient of variation.

^a^Approximate RNA copies/ml

^b^Cycle threshold (Ct) value

### Inter-assay precision

Variation of Ct values for inter-assay precision was generally greater than that observed for intra-assay precision. Percent CVs were lowest for YF17D-204, ranging from 0.3 to 1.05%. Percent CVs were higher for other prototype strains (614819, range 1.49 to 3.54%; Asibi, range 1.44 to 6.2%). Asibi displayed the highest inter-assay %CVs. Overall, the highest %CVs were observed in specimens possessing the lowest RNA concentrations (Table 2).

**Table 2:** Inter-assay precision of RealStar Yellow Fever Virus RT-PCR kit 1.0.

YF Strain	RNA copies/ml^a^	Operator 1^b^					Operator 2				Operator 3					Overall %CV
		1		2	3	4	1	2	3	4	1		2	3	4	
**17D-204 (Vaccine)**	**10** ^**6**^	28.3^c^	28.3		28.1	28.3	28.2	28.1	28.1	28.3	28.3	28.4		28.3	28.3	0.30
	**10** ^**5**^	31.2	31.1		31.2	31.1	31.0	30.9	30.8	30.9	31.0	31.1		31.0	30.9	0.41
	**10** ^**4**^	35.1	35.4		35.0	34.9	34.9	34.7	35.0	34.5	34.2	34.3		35.1	35.1	1.05
**614819 (Panama 1974)**	**10** ^**6**^	25.9	25.9		25.7	25.8	25.3	25.4	25.4	26.2	26.4	26.2		26.2	25.4	1.49
	**10** ^**5**^	29.0	29.9		29.8	29.8	29.3	29.3	29.2	28.3	28.7	29.7		29.7	29.5	1.69
	**10** ^**4**^	31.5	32.7		31.3	30.9	32.3	32.2	32.3	32.2	32.4	32.5		32.5	28.7	3.54
**Asibi (Ghana 1927)**	**10** ^**6**^	28.4	28.4		28.2	28.1	28.9	28.2	28.9	28.7	28.2	28.2		27.9	29.2	1.44
	**10** ^**5**^	31.4	31.3		30.2	31.3	30.1	31.4	31.4	31.4	31.3	31.6		30.0	29.2	2.53
	**10** ^**4**^	33.0	34.8		34.7	32.7	34.7	34.8	34.6	34.6	31.2	30.7		30.5	29.3	6.20

YF, yellow fever; RNA, ribonucleic acid; %CV, coefficient of variation.

^a^Approximate RNA copies/ml

^b^Three operators tested RNA extract in quadruplicate on 3 separate days

^b^Cycle threshold (Ct) value

### Inter-lot precision

Coefficients of variations of Ct values across two production lots of RealStar Yellow Fever Virus RT-PCR kit 1.0 were lowest for YF17D-204, ranging from 0.29 to 1.42%. Percent CVs were higher for other prototype strains (614819, range 0.65 to 1.89%; Asibi, range 1.26 to 3.37%). Observed variation was highest for the Asibi prototype virus, consistent with intra and inter-assay precision testing (Table 3).

**Table 3:** Inter-lot precision of RealStar Yellow Fever Virus RT-PCR kit 1.0.

YF Strain	RNA copies/ml^a^	Lot 1				Lot 2				Overall %CV
		1	2	3	4	1	2	3	4	
**17D-204**	**10** ^**6**^	28.3^b^	28.3	28.1	28.3	28.2	28.3	28.1	28.2	0.29
**(Vaccine)**	**10** ^**5**^	31.2	31.1	31.2	31.1	30.9	30.9	30.8	30.7	0.66
	**10** ^**4**^	35.1	35.4	35.0	34.9	34.3	34.0	34.7	34.2	1.42
**614819**	**10** ^**6**^	25.9	25.9	25.7	25.8	26.1	25.9	25.8	25.6	0.65
**(Panama 1974)**	**10** ^**5**^	29.0	29.9	29.8	29.8	28.9	29.1	29.1	29.8	1.50
	**10** ^**4**^	31.5	32.7	31.3	30.9	32.0	31.4	31.4	32.3	1.89
**Asibi**	**10** ^**6**^	28.4	28.4	28.2	28.1	28.4	30.3	29.9	30.3	3.37
**(Ghana 1927)**	**10** ^**5**^	31.4	31.3	30.2	31.3	31.2	31.2	31.4	31.3	1.26
	**10** ^**4**^	33.0	34.8	34.7	32.7	34.5	32.7	34.5	33.9	2.65

YF, yellow fever; RNA, ribonucleic acid; %CV, coefficient of variation.

^a^Approximate RNA copies/ml

^b^Cycle threshold (Ct) value

### Analytical sensitivity (LOD)

**Fig 1:**
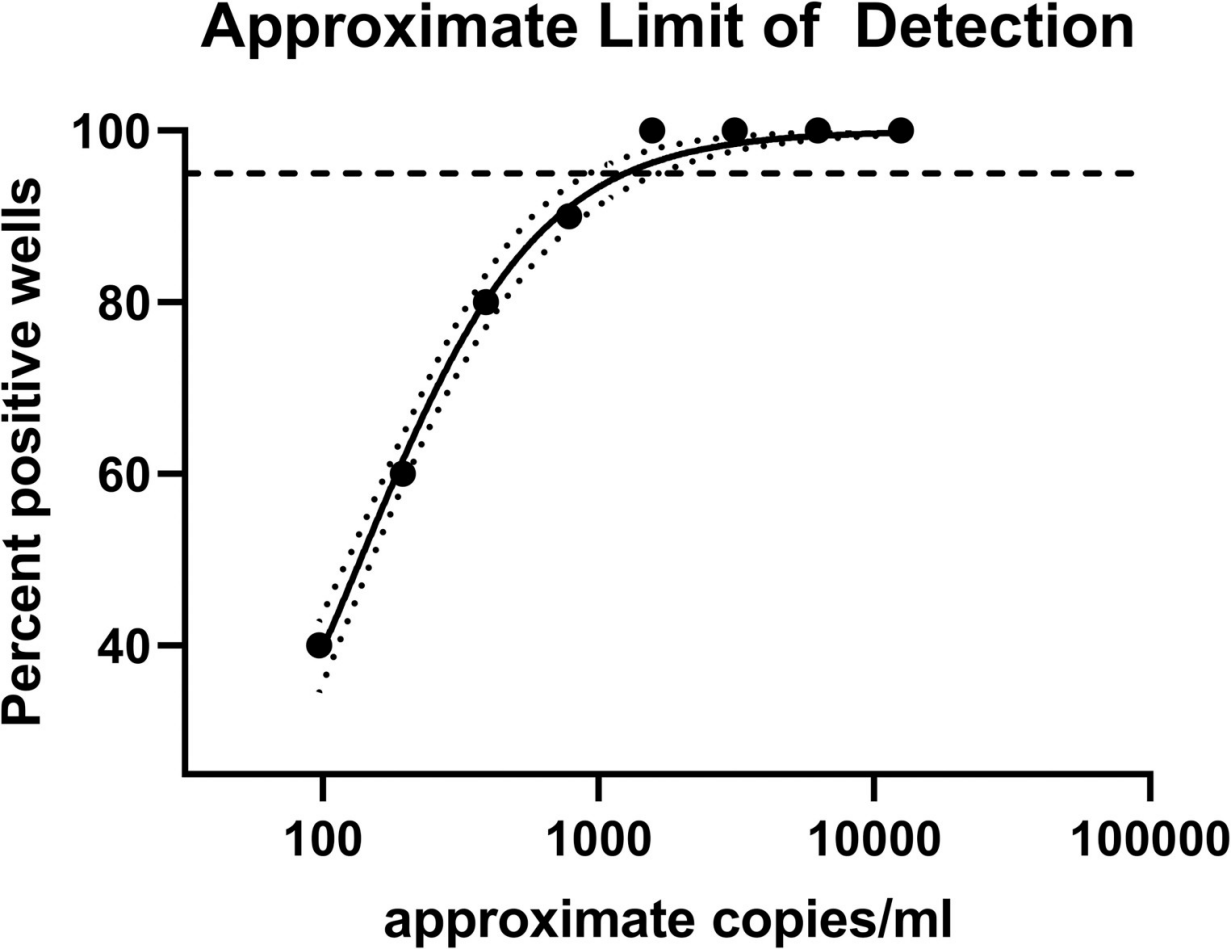
Approximate limit of detection of altona RealStar Yellow Fever Virus RT-PCR kit 1.0. Non-linear regression of the percent positive wells detected at approximate RNA concentrations. The dotted line on the y-axis depicts 95% detected wells. The curve-fitted dotted line indicates the 95% confidence range of the non-linear regression.

All replicates in the YF 17D RNA dilutions ranging from 25,000 to 1562 copies/ml gave positive results. Positivity of replicates dwindled at the lower dilutions: 9/10 (781 copies/ml); 4/5 (390.5 copies/ml); 3/5 (195 copies/ml) and 2/5 (97 copies/ml). The 95% LOD obtained was calculated to be approximately 1,245 copies/ml [95% confidence interval (CI) 497 to 1,640 copies/ml], which translates to 12.45 copies per 10 μl reaction. The CI contains the LOD claimed by the manufacturer [0.69 copies/μ1 or 690 copies/ml: 95% (CI) 410 to 1,560 copies/ml] (Fig 1).

### Inclusivity

The RealStar Yellow Fever Virus RT-PCR kit 1.0 successfully detected all YFV strains tested at all concentrations (Table 4).

**Table 4:** Inclusivity of RealStar Yellow Fever Virus RT-PCR kit 1.0.

YF Strain	RNA copies/ml^a^	Average Ct^b^
**17D-204**	**10** ^**6**^	28.25
**(Vaccine)**	**10** ^**5**^	31.17
	**10** ^**4**^	35.08
**614819**	**10** ^**6**^	28.25
**(Panama 1974)**	**10** ^**5**^	31.06
	**10** ^**4**^	33.78
**Asibi**	**10** ^**6**^	25.83
**(Ghana 1927)**	**10** ^**5**^	29.63
	**10** ^**4**^	31.59
**BA-55**	**10** ^**6**^	29.41
**(Nigeria 1986)**	**10** ^**5**^	32.36
	**10** ^**4**^	35.57
**BC-7914**	**10** ^**6**^	27.97
**(Kenya 1993)**	**10** ^**5**^	30.83
	**10** ^**4**^	34.32
**14FA**	**10** ^**6**^	27.55
**(Angola 1971)**	**10** ^**5**^	30.80
	**10** ^**4**^	34.61
**CAREC M2-09**	**10** ^**6**^	25.73
**(Trinidad 2009)**	**10** ^**5**^	29.84
	**10** ^**4**^	32.91
**FMD-1240**	**10** ^**6**^	25.70
**(Peru 2007)**	**10** ^**5**^	29.12
	**10** ^**4**^	31.66
**InHRR 10a-10**	**10** ^**6**^	25.34
**(Venezuela 2010)**	**10** ^**5**^	28.52
	**10** ^**4**^	32.63

YF, yellow fever; RNA, ribonucleic acid; Ct, cycle threshold

^a^Approximate RNA copies/ml

^b^Based on four replicates

### Analytical specificity

The RealStar Yellow Fever Virus RT-PCR kit 1.0 did not detect any of the 15 non-YF viruses tested.

### Interfering substances

When approximately 25,000 RNA copies/ml from YF 17D-204 were spiked into NHS containing bilirubin and triglycerides, Ct values like those obtained for 25,000 RNA copies/ml of 17D-204 in the analytical sensitivity experiments were observed. The spiked malaria antigen specimen yielded a lower average Ct value. This indicated that these substances had no adverse effects on YF RNA detection. High levels of hemoglobin in the contrived specimens as prescribed in the guiding document resulted in higher Ct values. Regardless, the RealStar Yellow Fever Virus RT-PCR kit 1.0 successfully detected YF RNA in specimens containing high levels of hemoglobin. (Table 5).

**Table 5:** The effects of potentially interfering substances on performance of RealStar Yellow Fever Virus RT-PCR kit 1.0.

Substance	Average Ct	%CV
**Triglycerides**	33.14	0.69
**Bilirubin**	32.29	2.8
**Hemoglobin**	35.84	0.72
**Malaria**	29.15	0.96
**Comparator** ^**a**^	33.02	0.02

CV, coefficient of variation; Ct, cycle threshold

^a^From 25,000 copies/ml Ct values obtained in analytical sensitivity experiments

### Clinical performance using surrogate specimens

Clinical sensitivity using surrogate specimens was found to be 100% with 95% CI 73.5–100%, and clinical specificity using surrogate specimens was found to be 100% with 95% CI 92.1–100%. One negative specimen from South America initially tested positive with a Ct value of 38.1. Upon re-extraction and re-testing this specimen was negative, and the original result determined to be from cross-contamination during RNA extraction.

### Platform comparison

Ct values generated on the Bio-Rad CFX96 and the ABI Prism 7500 Fast SDS differed by less than 1 cycle. The %CVs of Ct values were lower for the CFX96 (range, 0.05 to 1.26%) compared to the ABI7500 (range, 0.17 to 3.19%). Both instruments returned highest %CVs for the lowest concentration RNAs (Table 6).

**Table 6:** Comparison of precision values for the RealStar Yellow Fever Virus RT-PCR kit 1.0 using ABI Prism 7500 Fast SDS and Bio-Rad CFX96.

YF Strain	RNA copies/ml^a^	ABI Prism 7500 Fast SDS					Bio-Rad CFX96				
		1	2	3	4	%CV	1	2	3	4	%CV
**17D-204**	**10** ^**6**^	28.3	28.3	28.1	28.3	0.27	28.4	28.4	28.4	28.4	0.05
**(Vaccine)**	**10** ^**5**^	31.2	31.1	31.2	31.1	0.17	31.5	31.3	31.3	31.2	0.34
	**10** ^**4**^	35.1	35.4	35.0	34.9	0.60	35.9	35.1	35.1	35.0	1.26
**614819**	**10** ^**6**^	25.9	25.9	25.7	25.8	0.41	26.5	26.5	26.5	26.5	0.09
**(Panama 1974)**	**10** ^**5**^	29.0	29.9	29.8	29.8	1.45	29.9	29.9	29.8	29.9	0.20
	**10** ^**4**^	31.5	32.7	31.3	30.9	2.49	32.7	32.5	32.7	32.6	0.26
**Asibi**	**10** ^**6**^	28.4	28.4	28.2	28.1	0.57	28.6	28.6	28.5	28.6	0.17
**(Ghana 1927)**	**10** ^**5**^	31.4	31.3	30.2	31.3	1.80	31.3	31.4	31.5	31.6	0.41
	**10** ^**4**^	33.0	34.8	34.7	32.7	3.19	34.7	34.4	34.6	34.4	0.37

YF, yellow fever; RNA, ribonucleic acid; %CV, coefficient of variation

^a^Approximate RNA copies/ml

### Operational characteristics of the RealStar Yellow Fever Virus RT-PCR kit 1.0

The IFU and operational characteristics were assessed, and findings are listed in a standard form generated by WHO (S1 Table).

A table containing summarized performance data described here compared to the performance provided by altona Diagnostics to WHO for review, is available in a public document [23].

## Discussion

This brief, independent evaluation of the RealStar Yellow Fever Virus RT-PCR kit 1.0 revealed no notable differences in performance compared to the claims made by the manufacturer. The assay had lowest intra- and inter-assay, and inter-lot variations for YF17D-204 across all strains and concentrations, but all YF strains used with the assay in the precision analyses gave acceptable precision ranges for a qualitative molecular *in vitro* diagnostic assay according to ISO/FDIS 17822–2, section 7.3 [24]. No %CV maxima were identified in the TPP. The evaluations using YF (Asibi) gave %CVs generally close to the ranges reported by altona in the desktop review [23]. The between-lot precision indicated that lot variation would be minimal, although the lot investigation would have been more meaningful had more lots been evaluated.

The assay exhibited an analytical sensitivity similar to that described by Domingo et al. (2012) (1,245 copies/ml compared to 1,022 copies/ml for the reference assay) [20]. The approximate 95% CI of the LOD determined in the evaluation contained the LOD claimed in the IFU, so while a lower LOD was claimed by the manufacturer compared to the evaluation, the claim was reasonable. If the RNA used in this evaluation had been directly quantitated, rather than basing it off a pre-extracted quantity, the starting copy number may have been slightly higher, indicating that the evaluated and claimed LOD’s may have been even closer in value than reported. No maximum LOD was specified in the TPP, and while the guiding document listed 1,000 copies/ml as the upper acceptance limit for analytical sensitivity, this was ambitious based upon the reference assay. In addition, while no data was provided for inclusivity in the desktop review, the kit was shown here to be capable of detecting YF of multiple strains of geographically diverse origins, and thus can be expected to be useful in all YF-endemic regions. The RealStar Yellow Fever Virus RT-PCR kit 1.0 was designed to detect RNA from YF 17D vaccine virus. Positive results from recently vaccinated patients might occur, and must be investigated further to identify vaccine-related adverse events [25], or be interpreted in the context of the vaccination [14].

The RealStar Yellow Fever Virus RT-PCR kit 1.0 was found to be specific for YFV with respect to the viruses tested in this evaluation and we concluded the assay is unlikely to produce false-positive reactions with viruses closely related to YF virus, or from viruses eliciting similar clinical symptoms.

Contrived YF specimens containing potentially interfering substances commonly found in clinical specimens gave the expected YF-positive results with the RealStar Yellow Fever Virus RT-PCR kit 1.0, and thus it is probable that clinical specimens containing these substances will be capable of yielding positive results. While it was noted that higher Ct values were obtained for the specimens containing hemoglobin compared to the other substances that yielded Ct values consistent with those for 25,000 copies/ml in the LOD experiment, the concentration prescribed by the evaluation protocol (200 g/L) was 10-fold higher than the reported solubility of hemoglobin (20 g/L) and therefore this represented an unrealistic specimen surrogate with a thick and difficult-to-pipette homogenate.

The clinical performance study using surrogate specimens was intended to provide a brief survey of expected sensitivity and specificity. Data from a retrospective study of known positive and negative specimens was provided by altona Diagnostics for the desktop review based upon data from a collaborative study with FioCruz, Brazil. Clinical sensitivity and specificity for this kit was not listed in the IFU. For the desktop review, sensitivity was stated to be 100% with 95% CI (88.4–100%), based upon 30 YF-positive patient serum samples. The limited number of samples and origins did not represent a thorough analysis of clinical performance. Nonetheless, this compared favorably to the clinical sensitivity obtained in this evaluation when RNA was extracted from known negative clinical specimens from YF-endemic regions and spiked with YF virus.

A review of the IFU revealed that a Ct threshold to indicate positivity was absent, and any Ct was considered positive. A cutoff is an expected feature of molecular *in vitro* diagnostic assays. Follow-up discussions with altona resulted in plans for them to conduct an evaluation in collaboration with YF network laboratories to derive a real-world cutoff. The initial lot of kits used in this evaluation reached the expiration dates 3 months after receipt; however, data was available from the manufacturer to enable the expiration timeframe to be extended to a year from manufacture. This is now consistent with UNICEF guidelines for purchase and with the TPP [22].

There are several limitations to this analysis. True clinical pathogen-positive specimens were not available; thus, it was necessary to use contrived specimens. Acquiring good quality specimens obtained in a timely fashion following onset of symptoms is a major difficulty in some YF endemic areas. The interfering substances evaluation used specimens of simulated poor quality at a single virus concentration rather than true clinical specimens of poor quality or timing of acquisition. While the RNA copy number used in the simulated samples yielded Ct values consistent with those of patients who are just beginning to clear infections, this may not have been the best indication of how the assay performs under field conditions where specimens contain various concentrations of virus. In addition, the analytical specificity evaluation did not include non-viral etiologic agents. Only two real-time platforms were used to evaluate the kit although these are commonly used in YF laboratories.

The study was not intended as a thorough performance evaluation, but the results served the purpose of indicating whether the manufacturer’s performance claims were verifiable. The WHO LTWG reviewed these data and the desktop review report and concluded that performance data from altona Diagnostics was consistent with those of the independent evaluation, that the quality management system used for manufacture was satisfactory, and that the RealStar Yellow Fever Virus RT-PCR kit 1.0 was appropriate for use in YF-endemic regions.

A further expression of interest may be made by WHO to allow other manufacturers of YF molecular assays the possibility of being evaluated in a similar manner.

The introduction of an RT-PCR kit in resource-limited regions requires not only support for purchase of the kit itself, but for equipment, consumable supplies, and PCR accessories. Based upon the laboratory evaluation and desktop review, these items plus the RealStar Yellow Fever Virus RT-PCR kit 1.0 have been recommended for purchase and logistical support on behalf of countries at high risk of YF and eligible to receive Gavi funds. The lack of consistent reverse cold chain availability to ensure integrity of molecular specimens upon laboratory receipt in Africa presents an additional challenge. To address this, the EYE Strategy is facilitating, and Gavi is funding improved specimen transport.

Validation materials for the laboratories to perform in-house quality assurance are needed as well as training on the use of the kit. Specimens for use with in-laboratory validations are planned by the WHO LTWG in collaboration with the European Viral Archive and Robert Koch Institute, and workshops are in progress with altona Diagnostics GmbH staff serving as facilitators. A formal WHO external quality assurance exercise will be done at a future date that will serve as part of WHO laboratory accreditation for YF. This will inform the network regarding the success of the kit in these regions. While molecular testing is only appropriate for specimens acquired 10 days or less after onset of symptoms, and a negative result does not rule out infection, the recommendation and provision of a commercial, kit-based molecular assay for YF is expected to increase the availability of molecular testing for YF in Africa in particular. This should lead to a decreased reliance on serological methods and reduction in time to identify YF infections and outbreaks, a key goal of the EYE Strategy.

## Conclusion

This limited laboratory evaluation of assay performance showed the altona Diagnostics RealStar Yellow Fever Virus RT-PCR kit 1.0 to be appropriate for future molecular detection of YF viral RNA in YF-endemic regions. The introduction of the kit to African laboratories is expected to decrease YF outbreak detection times, and ultimately save lives.

## Supporting information

S1 Protocol WHO protocol for the performance evaluation of Yellow Fever Virus (YFV) molecular assays. (DOCX)

S1 Table Operational characteristics of the RealStar Yellow Fever Virus RT-PCR kit 1.0. (DOCX)
