# Supplementary material for: Laboratory evaluation of RealStar Yellow Fever Virus RT-PCR kit 1.0 for potential use in the global yellow fever laboratory network
Source: PLoS Negl Trop Dis. 2022 Sep 6;16(9):e0010770. doi: 10.1371/journal.pntd.0010770 (PMC9481164; doi:10.1371/journal.pntd.0010770)
Supplement: S1 Protocol — (DOCX) [file pntd.0010770.s001.docx]

WHO PROTOCOL
FOR THE PERFORMANCE
EVALUATION OF YELLOW FEVER Virus (YFV) MOLECULAR ASSAYS

World Health Organization 2020

**Table of Contents**

[Acknowledgements 3](#_Toc40197814)

[1. Introduction 4](#_Toc40197815)

[1.1 Qualification of Yellow Fever Virus (YF) molecular assays 4](#_Toc40197816)

[1.2 WHO Performance Evaluation of YF Nucleic Acid Tests 4](#_Toc40197817)

[2. Study Objectives 4](#_Toc40197818)

[2.1 Overall Objectives 4](#_Toc40197819)

[2.2 Specific Objectives: 4](#_Toc40197820)

[3. Study implementation 5](#_Toc40197821)

[3.1 Roles and Responsibilities 5](#_Toc40197822)

[3.2 Safety issues 6](#_Toc40197823)

[3.3 Transportation and Storage of Assays for Evaluation 6](#_Toc40197824)

[4. Study Design 6](#_Toc40197825)

[4.1 Precision of measurement 6](#_Toc40197826)

[4.2 Analytical Sensitivity 7](#_Toc40197827)

[4.3 Analytical Specificity 7](#_Toc40197828)

[4.4 Clinical Performance 7](#_Toc40197829)

[4.5 Usability Assessment 7](#_Toc40197830)

[5. Specimen Panels 7](#_Toc40197831)

[5.1 Specimen types 7](#_Toc40197832)

[5.2 Specimen Preparation 7](#_Toc40197833)

[5.3 Specimen Shipping and Transport 7](#_Toc40197834)

[5.4 Analytical Performance Specimen Reference Panel 8](#_Toc40197835)

[5.5 Clinical performance panel](#_Toc40197836) 8

[5.6 Criteria for eligible specimens](#_Toc40197836) 8

[6. Laboratory testing](#_Toc40197837) 10

[6.1 Review of the instructions for use](#_Toc40197838) 10

6.2 Analytical Performance Testing 10

[6.3 Analytical Sensitivity - Limit of detection (LoD)](#_Toc40197839) 10

[6.4 Analytical Specificity 1](#_Toc40197840)1

[6.5 Clinical Performance 1](#_Toc40197841)1

[6.6 Interpretation and recording of test results 1](#_Toc40197842)1

[7. Quality control 1](#_Toc40197843)1

[7.1 Test kit controls 1](#_Toc40197844)1

[7.2 External quality control specimen 1](#_Toc40197845)1

[7.3 Proficiency panels 1](#_Toc40197846)1

[7.4 Limits of acceptability 1](#_Toc40197847)1

[7.5 Interpretation of results 1](#_Toc40197848)2

[8. Analysis of data 1](#_Toc40197849)2

[8.1 Invalid runs 1](#_Toc40197850)2

[8.2 Invalid results (invalid internal quality control (IQC)/calibrator) 1](#_Toc40197851)2

[8.3 Analytical Sensitivity and Specificity 1](#_Toc40197852)2

[8.4 Clinical Performance 1](#_Toc40197853)2

[9. Report preparation 1](#_Toc40197854)2

[10. Materials and supplies 1](#_Toc40197855)3

11. Reference 13

[Annex 1 Assessment of operational characteristics 14](#_Toc40197856)

# Acknowledgements

The document WHO PROTOCOL FOR THE PERFORMANCE EVALUATION OF YELLOW FEVER (YF) MOLECULAR ASSAYS was produced under the coordination and supervision of Miguel Mulders, Scientist, Global VPD Laboratory Networks, WHO, Geneva, Switzerland with support from Gavi, the Vaccine Alliance. It was prepared in collaboration with Matthias Niedrig, Robyn Meurant, Anne-Laure Page, Lee Hampton, Jennifer Horton, Echemole Abass and Maurice Demanou and the YF Laboratory Working group.

# 1. Introduction

## 1.1 Qualification of Yellow Fever Virus (YFV) molecular assays

World Health Organization (WHO) YF Laboratory Network (YFLN) plays a pivotal role in the surveillance of yellow fever. WHO has chosen to apply a regulatory-based evaluation of commercial sources for molecular assays that detect virus RNA. This evaluation will interrogate the quality as well as the performance of these assays by assessment of the manufacturer’s quality management system, a review of critical verification and validation studies undertaken by the manufacturer (such as stability) and a laboratory-based performance evaluation.

## 1.2 WHO Performance Evaluation of YF Nucleic Acid Tests (NAT)

This document is intended to provide manufacturers of YF NATs and the WHO Evaluating Laboratory with the WHO performance evaluation procedure.

The protocol provides the details of the procedure for the laboratory-based performance evaluation of selected YF molecular assays (also referred to as nucleic acid tests and henceforth in this document as NATs) submitted for evaluation. It describes the procedures required to perform an evaluation of YF NATs submitted for evaluation. **This protocol is not intended to replace validation and verification studies that need to be conducted by the manufacturer.**

The evaluation verifies the performance characteristics of YF NATs, assessing their precision (reproducibility and repeatability), analytical sensitivity, and analytical specificity and clinical performance.

All assays will be performed using a commercially supplied quality control (QC) specimen, and a proficiency panel will be used by all analysts prior to performance evaluation.

Artificially prepared panels will be used to determine precision, inclusivity, analytical sensitivity and specificity. The clinical performance will be evaluated using specimens collected in a YF endemic region during a period of no outbreak. Given the non-availability of YF-positive serum specimens, clinical-like specimens will be generated by spiking serum with a range of concentrations of YFV grown in cell culture. In addition, operational characteristics of the assays will be evaluated, including ease of performance and suitability for use in laboratories within the YFLN.

Testing to evaluate the performance of the NATswill take place at a laboratory with recognised standards accreditation (traceable to ILAC)such as ISO 17025:2017 or ISO 15189:2012 for the performance of NATs.

# 2. Study Objectives

## 2.1 Overall Objectives

The overall objective of the protocol is to evaluate the performance of commercially available YF NATs undergoing WHO assessment.

## 2.2 Specific Objectives:

The specific objectives of the evaluation are to:

- Assess the precision of selected technologies
- Verify claims of limit of detection
- Assess interference and potential for cross-reactions
- Assess detection of different wild type YFV strains from Africa and South America
- Assess relative clinical performance using spiked and unspiked specimens collected during non-outbreak times from the clinical setting where the assay is intended to of used.
- Evaluate the operational characteristics of selected technologies, e.g. ease of performance, specimen type utility
- Report and disseminate the findings of the evaluation.

# 3. Study implementation

## 3.1 Roles and Responsibilities

The performance evaluation will be exclusively conducted by the WHO Evaluating Laboratory. The laboratory will be responsible for the following:

- Ensure availability of the specimen reference panels identified below and maintain their integrity and traceability
- Update specimen inventory
- Conduct testing for the performance evaluation, only using staff with proven competence in the assays under investigation, in accordance with internationally recognized best practices
- Prepare a draft report on laboratory evaluation results, including supervision of the analysis of the data with other YF Laboratory Network members
- Ensure all source data, data analysis records and all correspondence are retained and archived in a detectable manner, for a period of at least ten years
- In addition, the following aspects are key to minimising error and maximising the value of this performance evaluation.

The Principle Investigator of the WHO Evaluating Laboratory, will assume the following responsibilities:

- The Principle Investigator will be responsible for training the laboratory personnel on the details of the evaluation protocol and on the use of each assay undergoing evaluation
- Only those personnel who have received specific training for this evaluation will participate in the evaluation
- Accurate record keeping is crucial to the success of the evaluation and the Principle Investigator will be responsible for ensuring that all data generated by the evaluation are recorded on the agreed data collection sheets, and are accurate and up to date
- It is important to plan work in advance and follow standard operating procedures as prepared and controlled by the Evaluating Laboratory
- Before starting the test run, the operator will prepare worksheets and label all tubes, dilution vessels, test devices or plates with the specimen’s unique number, as appropriate
- It is recommended that any manual transcription involved in testing is checked for correctness by a second operator.

WHO and the YF Evaluation Working Group will assume the following responilibilities:

- Technical advice to the Principle Investigator
- Technical and administrative management of the laboratory evaluation
- Review and verification of the draft report, including seeking of comments on the draft from the manufacturer/s
- Preparation and dissemination of the final reports
- Formal, authorized contacts with the manufacturers
- Ensure publication of the results of these evaluations by WHO and any WHO recommendations derived from the evaluation(s) are be accompanied by the following disclaimer:

*“WHO and the WHO Evaluating Laboratory, do not warrant or represent that the evaluations conducted with the yellow fever NATs referred to in this document are accurate, complete and/or error-free. WHO and the WHO Evaluating Laboratory disclaim all responsibility for any use made of the data contained herein, and shall not be liable for any damages incurred as a result of its use. This document must not be used in conjunction with commercial or promotional purposes.”*

## 3.2 Safety issues

All patient samples could possibly contain other infectious agents like HIV, hepatitis B, C, D, G, E viruses as well as other potential causes of blood borne disease. Therefore, all types of specimens (whole blood, serum/plasma, body fluids, etc.) must be handled as potentially infectious. Appropriate precautions to minimize infectious hazards must be taken at all stages from the collection of specimens to the disposal of waste materials from the laboratory. The WHO Guidelines on HIV Safety Precautions, and Guidelines for the Safe Transport of Specimens (WHO/EMC/97.3) and the site’s guidelines on laboratory safety should be carefully followed by the laboratory staff.

## 3.3 Transportation and Storage of Assays for Evaluation

It is the responsibility of the manufacturer to ensure that the assays are received by the evaluating laboratory in good conditions, according to the instructions for transport and storage provided on the assays.

It is the responsibility of the WHO Evaluating laboratory to ensure all stated storage conditions are maintained after receipt of the assays into the laboratory. Daily records of temperatures shall be kept of all locations where test kits are received. The lot numbers of the test kits received/used, and their expiry dates are recorded on the individual run worksheets.

# 4. Study Design

The study will be conducted with the objective of verifying certain analytical performance characteristics. Although there are many performance characteristics that could be investigated, this evaluation is risk-based and focuses on aspects of greatest importance to assuring the safety and performance when such IVDs are used in WHO Member States.

Apart from where the YF NAT is part of a closed system, all specimens will be processed and tested using an extraction kit and thermocycler which are recommended by the manufacturer. Since the Qiagen QIAamp Viral RNA Mini Kit and the Applied Biosystems ABI 7500 are the extraction kit and thermocycler most commonly in place in the YF Laboratory Network, these should be part of the evaluation.

Analytical aspects that will be evaluated include the following performance characteristics:

## 4.1 Precision of measurement

- Intra-assay variation within a run (repeatability)
- Inter-assay variation between runs performed on three different days using different technicians (within-laboratory precision/reproducibility)
- Inter-lot variability based on testing of two independent lots of the product

## 4.2 Analytical Sensitivity

- Limit of detection (LoD)
- Inclusivity - Detection of African and South American wild type YFV strains

## 4.3 Analytical Specificity

- Reactivity with other flaviviruses
- Reactivity with other pathogens likely to be encountered in regions with YF.
- Reactivity with potentially interfering or inhibiting endogenous substances

## 4.4 Clinical Performance

- It is acknowledged that use of natural clinical specimens is preferable for a clinical evaluation.
- In the absence of known positive specimens available for testing, the clinical performance will consist of testing a series of YFV-negative clinical specimens spiked with YF virus in a randomized blinded fashion.

## 4.5 Usability Assessment

- Technicians with known skill in molecular assay performance will complete a questionnaire that include questions about the ease of use for the procedure including
- specimen and reagent preparation
- clarity of instructions for use
- record of any specific difficulties encountered or requirements

# 5. Specimen Panels

## 5.1 Specimen types

All panels will be prepared using the specimen type/s indicated in the NAT’s instructions for use. Where multiple specimen types are identified, serum will be the preferred specimen type. The clinical specimens will be serum collected from an endemic setting during a non-outbreak period. Where possible, specimens should be collected from patients with symptoms similar to those seen in YF.

## 5.2 Specimen Preparation

All personally identifying information of specimens used in the performance evaluation will be delinked from the specimen, with consequent labelling providing a link to results, not patient.

After removal of all the patient-specific identifiers, the specimens will be assigned a unique identification number for transport purposes and an agreed volume sent to the Evaluation Laboratory where they will be assigned the specimen identification number.

## 5.3 Specimen Shipping and Transport

Specimens are to be shipped to the evaluating laboratory using dry ice and will be stored at
-20°C (or preferable at -80°C if available) until use. During the period of testing the specimen aliquots will be stored at 4- 8°C for no longer than three days. To avoid frequent freeze/thaw cycles samples should be stored in aliquots. After the completion of testing, remaining volume of specimens will be stored at -20°C. Each aliquot should not undergo more than two freeze/thaw cycles. A system to monitor the sample handling shall be implemented.

## 5.4 Analytical Performance Specimen Reference Panel

Reference Panel preparation consists of cell culture-grown YF strains (17D YF vaccine strain, prototype African wild type strain, prototype South American wild type strain) diluted in YF negative serum.

The YF 17D standard preparation (RKI / European Virus Archive (EVA), <https://www.european-virus-archive.com/node/2495>) could be used as 17D reference material. Also the EQA YF Virus 195 panel from QCMD could be used as reference material. This panel comprises YFV 17D in different concentrations, Japanese encephalitis, West Nile, Zika, and Dengue 2 viruses for analysing the assay specificity.

- Precision of measurement:
- Three specimens representing the most relevant YFV strains (17D YFV vaccine strain, African wild type strain, South American wild type strain) will be used. Each YFV-positive specimen will be diluted in serum which has been tested negative for HIV, HBV and HCV to obtain a concentration of ca. 5000 ge copies / mL of the assay.
- Inclusivity:
- Specimens to demonstrate appropriate YF strain detection. They should comprise at least the 17D YFV vaccine strain, an African wild type strain and, a South American wild type strain. These specimens will be diluted to a concentration of 5000 ge copies/mL of the assay.
- Assessment of the Analytical sensitivity:
- The YFV 17D standard preparation from RKI will be used to prepare five-fold serial dilutions in YFV-negative serum comprising at least two concentrations at 5000 ge copies /mL, one concentration at 1000 ge copies /mL and two concentrations at 500 and 100 ge copies/ mL (Tab. 1)
- Analytical Specificity
- Non-YFV flavivirus strains and other pathogens (except malaria). Testing will be conducted with cell culture preparations of the various viruses (using prototype strains) spiked into negative serum specimens. Each virus will be spiked at concentrations found in clinical presentations.
- Interfering substances.
- Testing will be conducted with YFV 17D standard preparation at a concentration of ca. 10^3^ ge copies/mL spiked with challenging sera (see Table 1) in order to detect potential interference for the assays under evaluation. The malaria specimens will be those identified as positive by microscopy.

## 5.5 Clinical performance panel

- Left-over whole blood or serum submitted for routine testing can be used to evaluate the assays. Clinical specimens should be stored frozen at -20°C or preferable -80°C when available until testing. Total 100 specimens: 10 spiked with 5000 ge /mL of the African wild strain, 10 spiked with 5000 ge /mL of the South American wild strain and 80 non-reactive specimens will be tested.

## 5.6 Criteria for eligible specimens

- Specimens will be eligible for the evaluation study if they have a sufficient volume of ca. 1 mL serum to perform the assay under evaluation after all standard of care testing, including reference standard. The specimen must be of a type validated as suitable for use for the candidate assay.

### Table 1: Assay Performance Specimen Reference Panel

| **Analyte - Sample type** | **Concentration (copies/mL)** | **N° of replicates** | **Total N° replicates** |
| --- | --- | --- | --- |
| **Precision of measurement and inclusivity** |  |  |  |
| **Intra-assay precision/inclusivity** |  |  |  |
| YF 17D vaccine strain* | Each strain will be diluted at a concentration of ca. 10^3^, 10^4^ and 10^5^ ge copies /mL and run in quadruplicate | 3 | 12 |
| African wild type strain* |  | 3 | 12 |
| South American wild type strain* |  | 3 | 12 |
| **Inter-assay precision** |  |  |  |
| YF 17D vaccine strain* | Each strain will be diluted at a concentration of ca. 10^3^, 10^4^ and 10^5^ ge copies /mL and run in quadruplicate on 3 different days by different technicians (day 1 can use intra-assay data) | 3 | 24 |
| African wild type strain* |  | 3 | 24 |
| South American wild type strain* |  | 3 | 24 |
| **Inter-lot variability** |  |  |  |
| YFV 17D vaccine strain* | Each strain will be diluted at a concentration of ca. 10^3^, 10^4^ and 10^5^ and run in quadruplicate | 3 | 12 |
| YFV African wild type strain* |  | 3 | 12 |
| YFV South American wild type strain* |  | 3 | 12 |
| **Analytical Sensitivity** | | | |
| YFV Standard preparation* | ca. 10, 50, 100, 500, 1000, 5000, 10^4^, 5x10^4^, 10^5^, 5x10^5^, 10^6^ , 5x10^6^ ge copies /mL** | 5 | 25 |
| **Analytical Specificity (**Flavivirus strain detection) | | | |
| Hepatitis C* virus | Medium concentration  ca. 10,000 ge copies/mL | 1 |  |
| Dengue 1, 2, 3, 4*virus |  | 1 |  |
| Zika virus* |  | 1 |  |
| West Nile virus* |  | 1 | 9 |
| Tick Borne Encephalitis virus* |  | 1 |  |
| Japanese Encephalitis virus* |  | 1 |  |
| **Analytical Specificity (other pathogens)** | | | |
| Ebola virus* | medium concentration  ca. 10,000 ge copies/mL | 1 |  |
| Lassa fever virus* |  | 1 |  |
| Marburg virus* |  | 1 |  |
| HIV* |  | 1 | 8 |
| Hepatitis E virus* |  | 1 |  |
| Chikungunya virus* |  | 1 |  |
| Measles virus* |  | 1 |  |
| Influenza virus* |  | 1 |  |
| **Analytical Specificity (interfering substances)** | | | |
| Haemoglobin | 200 g/L | 3 |  |
| Triglyceride | 5.6 mmol/L | 3 | 12 |
| Bilirubin (unconjugated) | 257 µMol/L | 3 |  |
| Malaria positive sera |  | 3 |  |
| **Clinical Performance Panel** | | | |
| Non-reactive serum specimens Spiked African wild type | 5000 ge copies/ mL | 10 | 10 |
| Non-reactive serum specimens Spiked South American wildtype | 5000 ge copies/ mL | 10 | 10 |
| Non-reactive serum specimens |  | 80 | 80 |
| Total number of samples to be | tested |  | 305 |

ge = genome equivalent

* A inactivated YF prototypevirus grown in cellculture should be used for this evaluation.

All specimens will be prepared in concentrations representing the dynamic range of the assay under evaluation.

**Five of these dilutions will be chosen where the manufacturer’s LoD is approximately the middle dilution

# 6. Laboratory testing

## 6.1 Review of the instructions for use

Each product under evaluation will be used in accordance with manufacturer instructions. The evaluating laboaratory will send a copy of the instructions to WHO together with the evaluation report. Records of the instructions must be maintained as part of the evaluation report used must be kept.

**6.2 Analytical Performance Testing**

The determination of precision and inclusivity will be undertaken in a single set of experiments.

- Four specimens will be used: one YF negative, one YF 17D, one YF African wild type, and one YF South American wild type, at a concentration of 10^3^-10^5^ ge copies /mL, (see Table 1).
- Intra-run variability (repeatability): The same sample panel (ca.10^3^, 10^4^, 10^5^ ge copies/mL) should be tested on the same run. Each panel member should be tested in quadruplicate.
- Inter-run variability (reproducibility): The same sample panel (ca. 1000, 10^4^, 10^5^ ge copies/mL) should be tested in 3 different runs preferably by different technicians.Each panel member should be tested in quadruplicate on each run.
- Inter-lot variability: The same sample panel (ca.10^3^, 10^4^, 10^5^ ge copies/mL) should be tested on a second lot of the product. Tests should be performed in quadruple.

Results should not vary by more than 3 ct values, by frequent performance in the same laboratory.

Twelve concentrationss of YF 17D standard preparation, diluted as described in Table 1, will be tested 5 times. The 5 replicates will be separated in a minimum of three runs where applicable.

## 6.3 Analytical Specificity

For the evaluation of possible interfering substances for the assay performance different sera (malaria positive, lipaemic, hemolytic sera, see Table 1) will be spiked with ca.10,000 ge copies/mL 17D YF standard preparation. Tests should be performed in triplicate.

## 6.4 Clinical Performance

All specimens will be tested in singlicate in a randomized blinded fashion.

The acceptance criteria for the performance as 95% agreement at 1000 ge copies / mL, and 100% agreement at all other higher concentrations and for negative specimens.

## 6.5 Interpretation and recording of test results

The interpretation of results for each assay under evaluation is made according to the manufacturer’s instructions. All runs and/or test results are recorded on a data sheet.
Preferably all test results are recorded and exported directly from the instrument to standardized test result worksheets for further data analysis.

A technician's analysis is made of each assay under evaluation and is completed by the operator performing the testing. This analysis comprises operational characteristics, ease of the procedure, reading and interpretation of results, clarity of manufactural instructions, as well as records of any specific difficulties encountered during the evaluation. Special attention should be paid to the instruction leaflet regarding clarity, presentation, content and safety issues in order to evaluate whether these instructions are sufficient for diagnostic laboratories in developing countries.

# 7. Quality control

## 7.1 Test kit controls

Manufacturer-supplied positive and negative test controls will be run according to the manufacturer’s instruction. For assays without positive and negative controls provided by the manufacturer, the external quality control specimen will act as control.

Internal controls incorporated by the manufacturer into the assay are useful to verify the extraction, and/or amplification, and/or detection controls, as indicated in the manufacturer’s instruction.

## 7.2 External quality control specimen

The evaluating site will run a validated external control specimen. This external quality might be available from a commercial source (EVA, QCMD, see above). The 17D standard preparation in a medium (10,000 ge copies/mL) and low (1,000 ge copies/mL) concentration spiked in a YF negative test human serum should be used.

## 7.3 Proficiency panels

A proficiency panel of 10 specimens comprised of the three YFV strains (17D, African and South American wild type strain) at high (100,000 ge copies/mL), medium (10,000 ge copies/mL) and low (1,000 ge copies/mL) concentrations and serum spiked with media from uninfected cell culture (negative control);. This panel must be run successfully for each assay by each operator before the evaluation.

## 7.4 Limits of acceptability

All results on test kit controls and the external control specimen will be documented. If the control sample does not give the expected result, the run will be considered invalid, and the run will be repeated. All problems shall be recorded on a data sheet. The principle investigator will be responsible for carefully checking all data entry forms for legibility, accuracy and completeness.

## 7.5 Interpretation of results

The interpretation of results for each assay under evaluation is made according to the manufacturers’ instructions. Invalid test results and errors must be recorded. Recording will be done either directly via printer or manually in the data collection sheet. In order to avoid transcriptional errors, the four-eyes principle has to be performed. For safety reasons a digital record of the results will be kept.

# 8. Analysis of data

## 8.1 Invalid runs

The number of invalid test runs will be recorded as the absolute number of invalid runs and as a percentage of the total number of runs performed for the entire evaluation using all specimens.

## 8.2 Invalid results (invalid internal quality control (IQC)/calibrator)

The number of invalid specimens’ results is recorded. They are presented as a percentage of the total number of specimens tested per platform for the entire evaluation.

If applicable, other types of readings indicating an invalid result (e.g. error) will also be recorded and reported as a percentage of the total number of specimens tested of the entire evaluation.

## 8.3 Analytical Sensitivity and Specificity

The limit of detection (LoD) is the lowest concentration of analyte that can be consistently detected in at least 95% of specimens tested under routine laboratory conditions and in a given specimen matrix. It defines the analytical sensitivity. The sensitivity and LoD will be estimated using the Probit analysis on the results of the five replicates of the LoD panel.

An acceptance criteria for the lower performance limit of the assay shall be ≤1000 ge copies/mL. Assays that do not reach this LoD will not be considered for listing by the WHO.

The results of testing for each individual analyte should be reported separately. Where unexpected results have been encountered, these should be noted in the report

**8.4 Clinical Performance**

For the estimation of sensitivity and specificity of the assay under evaluation for the detection of YF nucleic acids, the results of the assay under evaluation will be compared to the expected result.

Specificity will be calculated as the proportion of true negative specimens identified by the index method compared to all negatives by the reference method. Specificity will be expressed as a percentage.

Sensitivity and specificity will be estimated with their 95% confidence intervals. The 95% confidence intervals are calculated in order to assess the level of uncertainty introduced by sample size. Exact 95% confidence intervals for binomial proportions will be calculated from the F-distribution.

The initial and re-testing results of specimens (undertaken in accordance with manufacturers instructions) will be described in the report.

# 9. Report preparation

WHO will produce a draft of the results, which will be shared with the evaluating laboratory and the manufacturer.

Manufacturers will have one-month right of reply. After one month the report will be accepted as final by WHO. The final report will be prepared and disseminated by WHO. A copy of the final report will be sent to the authorized contact designated by the manufacturer and to the laboratory.

# 10. Materials and supplies

Manufacturers will provide the products and any equipment necessary for the evaluation free of charge. According to Table 1 the number of tests to be performed for this evaluation is estimated at 310 single tests.

# 11. Reference

[Domingo C](https://www.ncbi.nlm.nih.gov/pubmed/?term=Domingo%20C%5BAuthor%5D&cauthor=true&cauthor_uid=23052311), [Patel P](https://www.ncbi.nlm.nih.gov/pubmed/?term=Patel%20P%5BAuthor%5D&cauthor=true&cauthor_uid=23052311), [Yillah J](https://www.ncbi.nlm.nih.gov/pubmed/?term=Yillah%20J%5BAuthor%5D&cauthor=true&cauthor_uid=23052311), [Weidmann M](https://www.ncbi.nlm.nih.gov/pubmed/?term=Weidmann%20M%5BAuthor%5D&cauthor=true&cauthor_uid=23052311), [Méndez JA](https://www.ncbi.nlm.nih.gov/pubmed/?term=M%C3%A9ndez%20JA%5BAuthor%5D&cauthor=true&cauthor_uid=23052311), [Nakouné ER](https://www.ncbi.nlm.nih.gov/pubmed/?term=Nakoun%C3%A9%20ER%5BAuthor%5D&cauthor=true&cauthor_uid=23052311),. Advanced yellow fever virus genome detection in point-of-care facilities and reference laboratories. J Clin Microbiol. 2012 Dec;50(12):4054-60. doi: 10.1128/JCM.01799-12. Epub 2012 Oct 10

# Annex 1. Assessment of operational characteristics

Date:

Name of assay       Name of operator

| General information | | | |
| --- | --- | --- | --- |
| 1 | What is the stated intended use of the assay? |  | |
| 2 | What is the principle of assay? |  | |
| 3 | What is the target of the assay? |  | |
| 4 | What is the region of amplification targeted by the assay? |  | |
| 5 | Which extraction method was used? Provide details | Automatic  Semi-automatic  Manual | |
| 6 | What is detection method of the final product? | End-point  Real-time | |
| 7 | What is the specimen volume required to perform the test? |  | |
| 8 | Which specimen types are compatible with the assay? | Serum  Plasma  Specify anticoagulant:  Whole blood  Other  Specify:  State the specimen type used: | |
| 9 | What is the test kit size evaluated (number of tests per kit)?  Which other test kit sizes are available? | tests/kit       tests/kit | |
| 10 | Controls included:  - Decontamination (Uracil-N-glycosylase [UNG])  - Positive (provided by manufacturer)  - Negative (provided by manufacturer)  - Internal controls (added to every specimen)  - External QC specimen + monitoring (provided by external source i.e. not the manufacturer) | YES  NO  YES  NO  YES  NO  YES  NO  YES  NO | |
| Operational aspects | | | |
| 11 | Are instructions for use concise and clear for:  the assay?  the extraction platform?  the amplification platform?  - What instruction language options are available? | | YES  NO  YES  NO  YES  NO  English  French |
| 12 | What is the total number of tests per run?  How many specimens can be tested per run?  How many controls are required per test run? | | Total Tests:  Patient Specimens:  Controls required: |
| 13 | What is the necessary time to obtain first result starting after specimen preparation. | | hours;      min |
| 14 | What is the hands-on time for the technician performing the assay? | | hours;      min |
| 15 | Technical skills required of staff:  -Reconstitution of reagents/buffers required  -Calculation of dilutions  -Number of steps required | | YES  NO  YES  NO  Number of steps: |
| 16 | How many days of training (by the manufacturer) are required to perform the assay? | | days |
| 17 | What is the rate of invalid runs during the evaluation period?  What is the rate of invalid individual results over the evaluation period? | |  |
| 18 | What is the level of automation of the platform, including the extraction component? | | |
| 19 | Having considered the elements above, assign a level of difficulty/complexity to the performance of the assay from specimen preparation to result (including analysis of the results):   \| Extremely Simple Very Complex \| \| \| \| \| \| --- \| --- \| --- \| --- \| --- \| \|  \|  \|  \|  \|  \| \| 1 \| 2 \| 3 \| 4 \| 5 \| | | |
| Reagents storage, specimen transport, processing and storage | | | |
| 20 | What is the shelf life upon manufacture of the reagents required to perform the assay? | | months |
| 21 | What is the claimed in-use stability of reagents once they have been opened? | | months |
| 22 | What are the specimen transport conditions required? | |  |
| 23 | Specimen processing procedure: | | |
| 24 | What are the specific storage conditions for :  -specimens?  -reagents?  -equipment? | | |
| Equipment and consumables | | | |
| 25 | Specify the dedicated equipment needs: | | |
| 26 | What general laboratory equipment is required to perform the assay but is not provided (e.g. vortex, waterbath, heating block, etc.)? | | |
| 27 | What are the reagents required to perform the assay but are not provided? (e.g. Fungicide, bleach, DNA/RNA decontamination products, ethanol): | | |
| 28 | Which laboratory consumables are required to perform the assay but are not provided (e.g. pipette tips, tubes, etc.)? | | |
| Infrastructure | | | |
| 29 | Dimensions & weight of equipment (metric system) | | - Extraction unit:  - Amplification:  - Detection unit: |
| 30 | What is the platform type? | | -Freestanding  - Bench top  -Other: |
| 31 | What are the dimensions of test kit (reagent pack)? | |  |
| 32 | Infrastructure (mechanical/power, physical, environmental)  -What are the voltage requirements?  UPS, brownouts, blackouts  - Is a temperature controlled room (air-conditioned) required?  -Is a dust-free environment required?  -Are there any altitude or humidity specifications?  -Distilled/deionised water requirements | | Voltage:  YES  NO  YES  NO  Altitude:       Humidity: |
| 33 | What are the requirements for separation of workspace? (e.g. specimen processing, extraction, amplification) | |  |
| Waste disposal and biosafety | | | |
| 34 | What are the waste volumes produced per run?  Does disposal of the consumable pose a substantial infection risk? | | Volume:  YES  NO |
| 35 | Are there any safety concerns for the user?  -Biological hazard (consider specimen collection, specimen processing eg centrifugation, hardware contamination, aerosol production, wastes)  -Chemical hazards (e.g. for extraction methods): | | YES  NO  YES  NO  YES  NO  Specify: |
| Calibration, maintenance and troubleshooting | | | |
| 36 | Is daily calibration of the instrument necessary? | | YES  NO  If answer is NO, specify frequency: |
| 37 | What is the maintenance frequency required for the equipment? (e.g. Fungicide treatment, bleach treatment, RNA decontamination, ethanol, UV-irradiation) | | Daily  Weekly  Monthly  Yearly  No need |
| 38 | What was the frequency of breakdown/blockage over the study period? | | None  1-5 times  > 5 |
| 39 | What was the response time of the instrument technician/engineer after notification of the instrument's breakdown? | | Same day  1-2 days  3-5 days  >5 days |
| Data Management | | | |
| 40 | What is the system for data collection and storage?  and compatibility/interfacing with LIMS | | |
| 41 | Is a printer required? | | YES  NO  If YES, is it provided? YES  NO |
| 42 | What language options are available for the software and results? | | English  French |
| Final Appraisal, Comments | | | |
| 43 | Having considered all the elements above, does the technician in charge of the evaluation consider the assay to be   \|  \|  \|  \|  \|  \| \| --- \| --- \| --- \| --- \| --- \| \| 1 \| 2 \| 3 \| 4 \| 5 \| \| Poor \| Needs Improvement \| Satsfactory \| Good \| Excellent \| | | |
| 44 | What are the advantages of this platform?    What are the disadvantages of this platform?    Other comments: | | |
