# Supplementary material for: Laboratory evaluation of RealStar Yellow Fever Virus RT-PCR kit 1.0 for potential use in the global yellow fever laboratory network
Source: PLoS Negl Trop Dis. 2022 Sep 6;16(9):e0010770. doi: 10.1371/journal.pntd.0010770 (PMC9481164; doi:10.1371/journal.pntd.0010770)
Supplement: S1 Table — (DOCX) [file pntd.0010770.s002.docx]

# Annex 1. Assessment of operational characteristics

**Part of Report of the Evaluation of** **Altona RealStar® Yellow Fever virus RT-PCR 1.0**

Date: 1-7-2021

Name of assay: **Altona RealStar® Yellow Fever virus RT-PCR 1.0** Name of operator: Holly Hughes

| General information | | | |
| --- | --- | --- | --- |
| 1 | What is the stated intended use of the assay? | The RealStar® Yellow Fever Virus RT-PCR Kit 1.0 is an *in vitro* diagnostic test, based on real-time PCR technology, for the qualitative detection of yellow fever virus specific RNA. | |
| 2 | What is the principle of assay? | Real-time RT-PCR technology utilizes reverse-transcriptase (RT) reaction to convert RNA into complementary DNA (cDNA), polymerase chain reaction (PCR) for the amplification of specific target sequences and target specific probes for the detection of the amplified DNA. The probes are labelled with fluorescent reporter and quencher dyes. | |
| 3 | What is the target of the assay? | Yellow fever viral RNA and internal control RNA | |
| 4 | What is the region of amplification targeted by the assay? | Gene target(s) not specified | |
| 5 | Which extraction method was used? Provide details | Automatic  Semi-automatic  Manual  Qiagen QIAamp Viral RNA mini kit | |
| 6 | What is detection method of the final product? | End-point  Real-time | |
| 7 | What is the specimen volume required to perform the test? | Original serum: 140ul for RNA extraction (following extraction protocol)  RNA: 10ul per well | |
| 8 | Which specimen types are compatible with the assay? | Serum  Plasma  Specify anticoagulant:  Whole blood  Other  Specify: “extracted RNA” p.13 IFU  State the specimen type used: extracted RNA from spiked serum | |
| 9 | What is the test kit size evaluated (number of tests per kit)?  Which other test kit sizes are available? | 96 tests/kit  NA tests/kit | |
| 10 | Controls included:  - Decontamination (Uracil-N-glycosylase [UNG])  - Positive (provided by manufacturer)  - Negative (provided by manufacturer)  - Internal controls (added to every specimen)  - External QC specimen + monitoring (provided by external source i.e. not the manufacturer) | YES  NO  YES  NO  YES  NO  YES  NO  YES  NO | |
| Operational aspects | | | |
| 11 | Are instructions for use concise and clear for:  the assay?  the extraction platform?  the amplification platform?  - What instruction language options are available? | | YES  NO  YES  NO  YES  NO  English  French  Portugese, Spanish, Italian |
| 12 | What is the total number of tests per run?  How many specimens can be tested per run?  How many controls are required per test run? | | Total Tests: 96  Patient Specimens: 94  Controls required: 2 (IFU: 1 positive and on negative) |
| 13 | What is the necessary time to obtain first result starting after specimen preparation. | | 2hours; 45 min |
| 14 | What is the hands-on time for the technician performing the assay? | | hours; 45 min |
| 15 | Technical skills required of staff:  -Reconstitution of reagents/buffers required  -Calculation of dilutions  -Number of steps required | | YES  NO  YES  NO  Number of steps: reaction and plate set up: 4  Instrument set-up: 10  Interpretation: 5 |
| 16 | How many days of training (by the manufacturer) are required to perform the assay? | | 0 days |
| 17 | What is the rate of invalid runs during the evaluation period?  What is the rate of invalid individual results over the evaluation period? | | 0/17  0/531 |
| 18 | What is the level of automation of the platform, including the extraction component? None (however, automated extraction systems approved by manufacturer) | | |
| 19 | Having considered the elements above, assign a level of difficulty/complexity to the performance of the assay from specimen preparation to result (including analysis of the results):   \| Extremely Simple Very Complex \| \| \| \| \| \| --- \| --- \| --- \| --- \| --- \| \|  \|  \|  \|  \|  \| \| 1 \| 2 \| 3 \| 4 \| 5 \| | | |
| Reagents storage, specimen transport, processing and storage | | | |
| 20 | What is the shelf life upon manufacture of the reagents required to perform the assay? | | Manufacturing date not given on box. At time of receipt of kits, there were 90 days of shelf-life before expiration |
| 21 | What is the claimed in-use stability of reagents once they have been opened? | | No more than two freeze thaw cycles. Shelf-life in months not given. |
| 22 | What are the specimen transport conditions required? | | None stated in IFU |
| 23 | Specimen processing procedure:  Seven manual or automated RNA extraction methods have been validated by manufacturer (IFU p. 13). | | |
| 24 | What are the specific storage conditions for :  -specimens? Not indicated  -reagents? Frozen -15C to -25C  -equipment? None indicated. States refer to appropriate manufacturer’s information | | |
| Equipment and consumables | | | |
| 25 | Specify the dedicated equipment needs:  As listed in the IFU:   1. Appropriate real-time PCR instrument (see chapter 6.1 Real-Time PCR Instruments) 2. Appropriate nucleic acid extraction system or kit (see chapter 8.1 Sample Preparation) 3. Desktop centrifuge with a rotor for 2 ml reaction tubes 4. Centrifuge with a rotor for microtiter plates, if using 96 well reaction plates 5. Vortex mixer 6. Appropriate 96 well reaction plates or reaction tubes with corresponding (optical) closing material 7. Pipettes (adjustable) 8. Pipette tips with filters (disposable) 9. Powder-free gloves (disposable) 10. Not listed in IFU: 11. Laptop or desktop computer for PCR instrument analysis | | |
| 26 | What general laboratory equipment is required to perform the assay but is not provided (e.g. vortex, waterbath, heating block, etc.)?  As listed in IFU:   1. Desktop centrifuge with a rotor for 2 ml reaction tubes 2. Centrifuge with a rotor for microtiter plates, if using 96 well reaction plates 3. Vortex mixer   Pipettes (adjustable)  Not listed in IFU:  Cold blocks | | |
| 27 | What are the reagents required to perform the assay but are not provided? (e.g. Fungicide, bleach, DNA/RNA decontamination products, ethanol):  Not indicated in IFU, however decontamination (e.g. bleach, lysol), and DNAse/RNAse and DNA contamination removal solutions would be recommended. | | |
| 28 | Which laboratory consumables are required to perform the assay but are not provided (e.g. pipette tips, tubes, etc.)?   1. As listed in IFU: 2. Pipette tips with filters (disposable) 3. Powder-free gloves (disposable) | | |
| Infrastructure | | | |
| 29 | Dimensions & weight of equipment (metric system) | | - Extraction unit: 33 cm W x 22.8 cm H x 33.02 cm D (benchtop centrifuge)  - Amplification:  - Detection unit: Note that both require computer  ABI: 34 cm (W) x 49 cm (H) x 45 cm (D)  CFX: 34 cm W x 38.1 cm H x 45.7 cm D |
| 30 | What is the platform type? | | -Freestanding  - Bench top  -Other: |
| 31 | What are the dimensions of test kit (reagent pack)? | | Box: 5.5 in W x 3.5 in H x 4.5 in D |
| 32 | Infrastructure (mechanical/power, physical, environmental)  -What are the voltage requirements?  UPS, brownouts, blackouts  - Is a temperature controlled room (air-conditioned) required?  -Is a dust-free environment required?  -Are there any altitude or humidity specifications?  -Distilled/deionised water requirements | | For ABI7500:  Voltage: 100-240  UPS recommended by manufacturer  YES  NO 15-30°C  YES  NO  Altitude:       Humidity: 20-80%  No DI water listed in IFU or manufacturer recommendations |
| 33 | What are the requirements for separation of workspace? (e.g. specimen processing, extraction, amplification) | | Use separated and segregated working areas for (i) sample preparation, (ii) reaction setup and (iii) amplification/detection activities. |
| Waste disposal and biosafety | | | |
| 34 | What are the waste volumes produced per run?  Does disposal of the consumable pose a substantial infection risk? | | Volume: 30 ul of liquid waste per well. One 96-well plastic reaction plate per run.  YES  NO |
| 35 | Are there any safety concerns for the user?  -Biological hazard (consider specimen collection, specimen processing eg centrifugation, hardware contamination, aerosol production, wastes)  -Chemical hazards (e.g. for extraction methods): | | YES  NO  YES  NO  YES  NO  Specify: guanidinium thiocyanate (AVL buffer) |
| Calibration, maintenance and troubleshooting | | | |
| 36 | Is daily calibration of the instrument necessary? | | YES  NO  If answer is NO, specify frequency:  yearly |
| 37 | What is the maintenance frequency required for the equipment? (e.g. Fungicide treatment, bleach treatment, RNA decontamination, ethanol, UV-irradiation) | | Daily  Weekly  Monthly  Yearly  No need |
| 38 | What was the frequency of breakdown/blockage over the study period? | | None  1-5 times  Door malfunction 1 time on ABI 7500  > 5 |
| 39 | What was the response time of the instrument technician/engineer after notification of the instrument’s breakdown? | | Same day  1-2 days  3-5 days  >5 days |
| Data Management | | | |
| 40 | What is the system for data collection and storage? Manufacturer provided computer, compatibility with LIMS unknown  and compatibility/interfacing with LIMS | | |
| 41 | Is a printer required? | | YES  NO  If YES, is it provided? YES  NO |
| 42 | What language options are available for the software and results? | | English  French  Unknown if other languages available |
| Final Appraisal, Comments | | | |
| 43 | Having considered all the elements above, does the technician in charge of the evaluation consider the assay to be   \|  \|  \|  \|  \|  \| \| --- \| --- \| --- \| --- \| --- \| \| 1 \| 2 \| 3 \| 4 \| 5 \| \| Poor \| Needs Improvement \| Satsfactory \| Good \| Excellent \| | | |
| 44 | **What are the advantages of this platform?**  Easy to use kit (two tubes of master mix, one tube of internal control). No need for dilutions/calculations of primers and probes. 96 reactions provided in aliquots to avoid freeze-thaw cycle degradation. Specificity, LOD, and precision were found to be similar to manufactuerer’s claims.  **What are the disadvantages of this platform?**  No Ct cycle cut-off given for interpretation of positive vs negative. All positive amplification is considered positive. Evaluating amplification curves after 40 cycles is difficult to determine if true logarithmic amplification is occurring or background “noise.” Also, we had two instances of contamination both of which were positive >38 cycles. Re-extraction confirmed these samples as negative. Therefore without a cut-off Ct value, results with high Ct values should be interpreted with caution and perhaps re-extracted for confirmation.  **Other comments:**  Limit of detection: approximately 1,245 c/ml  Unusual temperature, cycling conditions and quenchers requires attention to detail by the technician setting up the software.  BioRad CFX96 is more user friendly and has more straightforward software/set-up. Unusal quencher requirements are negated with CFX96 as the quencher does not effect the results. Assigning of an inappropriate quencher on the ABI7500 software resulted in detrimental signal. | | |
